# Korean adolescent suicide and search volume for “self-injury” on internet search engines

**DOI:** 10.3389/fpsyt.2023.1186754

**Published:** 2023-06-06

**Authors:** Jin Yeong Son, Jun Hee Han, Seung Chan Kim, Won-Seok Choi, Hyun Ju Hong

**Affiliations:** ^1^Department of Psychiatry, Hallym University Sacred Heart Hospital, Anyang, Republic of Korea; ^2^Department of Psychiatry, Hallym University Sacred Heart Hospital, College of Medicine, Hallym University, Anyang, Republic of Korea; ^3^Department of Biostatistics, Clinical Trial Center, Biomedical Research Institute, Pusan National University Hospital, Busan, Republic of Korea; ^4^Department of Psychiatry, Yeouido St. Mary’s Hospital, College of Medicine, The Catholic University of Korea, Seoul, Republic of Korea; ^5^Hallym University Suicide and School Mental Health Institute, Anyang, Republic of Korea

**Keywords:** adolescent, suicide, internet search, self-injury, sex difference, suicidal attempt

## Abstract

**Introduction:**

Many adolescents with suicidal ideation receive support through the Internet. However, they also find ways to attempt suicide or strengthen their suicidal ideation through this medium. This study analyzed the association between the search volume of suicide-related terms and the number of suicides among Korean adolescents. We also analyzed the correlations between the search volumes of suicide-related terms.

**Methods:**

We selected seven words (suicide, self-injury, depression, academic score, school violence, outcasts, and family trouble) related to adolescent suicide. A dataset was constructed by combining data from the most commonly used search engine in Korea (Naver Datalab) and the daily number of adolescent suicides in school settings (*n* = 347) from January 1, 2016 to December 31, 2018, collected from the Ministry of Education. Poisson regression and Pearson correlation analyses were performed.

**Results:**

Significant associations were found between suicide attempts and search term volumes, which differed according to sex and time interval. Among the search terms, “self-injury” was most strongly associated with suicide, and this association was significant at all time intervals (daily, weekly, and monthly) in female adolescents and in the total population. Further, the association was strongest in the daily suicide data. More search term volumes were related to suicide in the daily and weekly data than in the monthly data. There were positive correlations between “suicide,” “self-injury,” and “depression” search volumes.

**Conclusion:**

Further studies with larger sample sizes, more search terms, and analysis of time intervals between suicide-related term search and suicide death are required. These studies can contribute to the establishment of an online suicide prevention system to detect suicide risk in adolescents and provide interventions.

## 1. Introduction

In the modern society, the Internet is an indispensable tool inextricably linked to real life; information is obtained and interpersonal relationships are built and maintained through the Internet. Adolescents are more familiar and comfortable with the Internet than adults. Suicide is a serious social problem and a leading cause of death among Korean adolescents (1). The rate of death by suicide per 100,000 individuals has decreased or remained constant in most Organization for Economic Cooperation and Development (OECD) countries (2). In contrast, in Korea, suicide-attributed deaths increased by 46%, and suicide rates were higher (24.6 per 100,000 individuals) than those in other countries from 2000 to 2019 (3). The suicide rate among Korean adolescents tends to increase with age, and the difference in rates between male and female adolescents is lower than that in other OECD countries. The sex ratio is not higher than 2:1, and the suicide rate of female adolescents is relatively higher with a rising trend (4, 5). Many adolescents with suicidal ideation (SI) receive support through the Internet (6, 7). Using the information they obtain from Internet searches, they become empowered to attempt suicide or acquire information on methods of attempting suicide (8–10). Problematic Internet use in adolescents is known to be associated with psychiatric symptoms (11), SI, and depression (12). This suggests that adolescents with mental health problems, such as depression, may be engaging in problematic Internet use.

Internet searches are an indicator of social activity and have been explored as a new source of suicide risk monitoring (13–17). A recent study (16) indicated the possibility of detecting SI through social media. Further, data derived from the Spanish Twitter shows high accuracy in predicting suicide risk (17). A United Kingdom analysis based on Google Trends data showed a link between the Internet search volume of suicide-related terms and suicide mortality, especially in men under 34 years of age (18). Studies conducted in Taiwan (19) and Japan (20) found an association between the search volumes of “depression” and suicide deaths, while another study indicated that search volumes for specific suicide methods, such as “hydrogen sulfide use,” were related to suicide death (21). Conversely, a Spanish study using Google Trends data showed a negative correlation between the Internet search volume of suicide-related terms and suicide deaths (22). Furthermore, search terms related to suicide deaths differ according to sex. A study in Taiwan (19) reported that the search volume of “depression” was related to suicides among males but not among females. In contrast the search volume of “anxiety disorder” was related to suicides among females but not among males. In a study conducted in the United States, suicide-related deaths and the search volume for the term “suicide” showed a significant negative correlation in the general population, while the search volumes for the terms “suicide” and “self-harm” showed significant positive correlations with suicide-related deaths among 18 to 25 year-olds (23).

Adolescents exhibit distinct activity patterns compared to adults and are more adept at using the Internet, often relying on it as a primary source of health-related information (24). Additionally, adolescent patients with psychiatric conditions searched for suicide- or self-harm-related terms more frequently than adult patients (25). Thus, in adolescents, the associations between suicide-related term search volumes and suicide attempts (SA) may be more significant than those in adults. However, to date, studies on this subject have focused on adults (14, 19–22), and adolescents have not been analyzed separately (15, 18, 23).

Adolescent suicides increased rapidly during the first 10 days after the media reported a celebrity suicide (26, 27), and the age of impulsive suicide attempters is younger than that of non-impulsive suicide attempters (28). These results suggest that the association between Internet searches and suicide-related deaths may be as short as 1 month. However previous studies used Google Trends data, which provides a minimum unit of search volume at weekly intervals and does not provide information concerning user sex. Moreover, the shortest time interval between the volume of search terms and suicide was 1 month (14, 19–22).

Naver^1^ is the most commonly used Internet search engine in Korea, representing the basic choice for most Korean users. More elaborately, based on a recent survey, this search engine receives approximately 30 million visitors daily and is estimated to be used by approximately 76% of Korean Internet users as the main search portal (29, 30). In previous studies, the Google search engine was used, which accounted for a significant portion of the search market in the United States, most European countries, and Japan as of February 2010, with market shares of 80.97, 94.85, and 78.47%, respectively (31).

Naver DataLab^2^ provides search volume data and trends of specific terms at least daily. This allows for a more precise analysis compared to using Google’s search volume units, which are measured on a weekly basis.This study combined Naver Datalab data with the total student suicide data from the Ministry of Education of Korea to reveal possible relationships between suicide deaths and related word Internet searches in adolescents (13 to 18 year-olds). The data included the date of death by suicide.

This study had two goals. First, we aimed to identify associations between the search volumes of suicide-related search terms and suicide deaths in Korean adolescents according to sex and time interval. Second, we sought to determine the correlations between the search volumes of suicide-related search terms.

## 2. Materials and methods

### 2.1. Database

This study is based on student suicide reports collected by the Korean Ministry of Education from January 1, 2016, to December 31, 2018. When a student dies of suicide, the school must report relevant information to the Ministry of Education using a student suicide report form. Details of the student suicide report, including the date of death, have been described previously (5). If the date of death is unknown, the date the body was found was regarded as the date of death. In approximately 70% of Korean adolescents who attempt suicide, the chosen suicide method is falling from a height (32). Thus, the time between the fall and death is estimated as short.

In Korea, education up to middle school is compulsory, and the dropout rate of high school students in 2021 was 1.5% (33). Thus, this data may reflect several general characteristics of suicide among children and adolescents in Korea. We extracted the date of death of students who died of suicide (*n* = 366). Of these, 347 students aged 13 to 18 years were included in the study.

We selected seven search terms related to suicide based on previous studies (14, 15, 18–23) and Korean studies on risk factors of suicide (5, 32). The suicide-related search terms were “Suicide” (“자살” in Korean), “Self-Injury” (“자해” in Korean), “Depression” (“우울” in Korean), “Academic Score” (“성적” in Korean), “School Violence” (“학교 폭력” in Korean), “Outcast” (“왕따” in Korean), and “Family Trouble” (“가정 불화” in Korean). For “Family Trouble,” search volume trends for sub-terms, “Divorce” (“이혼” in Korean), and “Domestic Violence” (“가정 폭력” in Korean), were added.

“Outcast” is a term referring to a form of school violence and involves group bullying. This form of bullying by isolating an individual from their peers is common in Korea. Since this term is representative, it was selected as a separate search term from “School Violence.”

Data on the volumes of these search terms were obtained from Naver Datalab^3^ on June 16, 2019. Naver Datalab provides search trends using the relative search volume (RSV) for each term. The RSV ranges from 0 to 100, corresponding to the number of searches for a given term as a proportion of the largest number of searches over a given period. For example, an RSV of 50 equates to 50% of the most popular search activity at any time. While Google Trends provides average RSVs over at least 1 week without providing information on sex, Naver Datalab extracts the daily search volume of a given term, providing information on the age and sex of logged-in users. The daily RSVs of seven suicide-related search terms for adolescents aged 13 to 18 years were obtained from January 1, 2016, to December 31, 2018. RSVs were also obtained separately according to sex. The search volumes in this study include all searches performed on personal computers and mobile devices.

This study was approved by the institutional review board of Hallym University Sacred Heart Hospital (approval number: 2020-08-008). Written informed consent from the participants’ legal guardian/next of kin was not required for participation in this study in accordance with national legislation and institutional requirements.

### 2.2. Statistical analysis

A dataset was constructed by combining data from the Naver Datalab and the daily numbers of adolescents who committed suicide from January 1, 2016, to December 31, 2018 for statistical analysis.

Through basic visualization, the distribution of suicide by point of time was identified, and missing values were estimated using linear interpolation, considering the time series tendency.

We employed a multivariate Poisson regression model to reveal associations between the total suicides per day and the volumes of search terms. Previous studies (19–21) that analyzed associations between suicide-related search terms and the suicide rate showed a different time lag between the volumes of search terms and suicide rate. Considering this time lag, a Poisson regression analysis of the number of suicides and search volumes was performed at different time intervals. The daily search volume of each term was summed to generate weekly and monthly search volumes, and possible associations between the search volumes and number of suicides were analyzed. We used the volumes of the seven search terms as independent variables and the number of suicides as dependent variables. Univariate analysis was performed, and multivariate analysis was performed with variables found to be statistically significant in the univariate analysis.

The correlations between Naver Data Lab search terms were examined using Pearson’s correlation analysis for male adolescents, female adolescents, and all subjects. R software was used for statistical analyses.

## 3. Results

### 3.1. Trends in search term volumes and the number of suicides on a weekly basis

**Figure 1:**
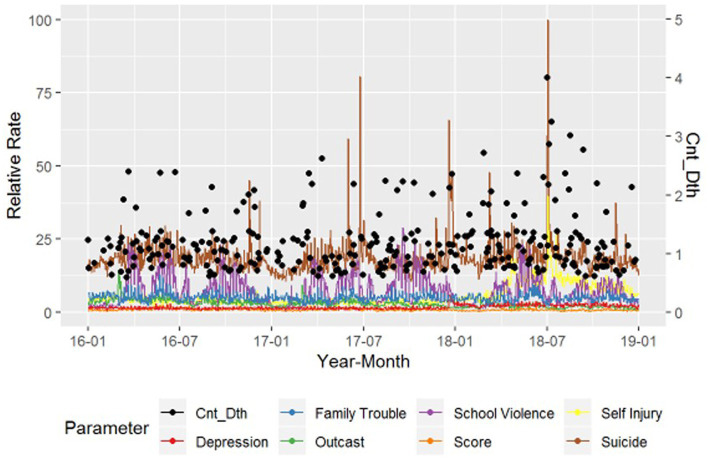
Number of suicides per day and the relative rate of each search term volume by period. Cnt_Dth: number of suicides per week. Relative rate: relative rate of each search volume from Naver Datalab.

Figure 1 shows the number of suicides recorded from January 2016 to December 2018 and the RSV of each search term. The suicide rate reached the highest value in 2018, and the search volume for “Self-Injury” increased compared to previous years. The search volume for “Suicide” increased rapidly at a point in time, while the search volume for other words was relatively small.

The number of suicides and search volumes of the terms “Suicide” and “Self-Injury” in 2018 was higher than those in other years that were examined in our study. In 2018, adolescent self-injury emerged as a social problem in Korea. The same year, the suicide rate in Korea among those aged 10 to 19 years was 5.8 per 100,000 individuals (*n* = 300), reflecting a 23% increase compared to the respective rate of that was documented the previous year (4.7%; *n* = 254). Of interest, the female suicide rate (5.9%) was higher than the male suicide rate (5.7%), which is unusual (34).

### 3.2. Distribution of the number of suicides per day

**Figure 2:**
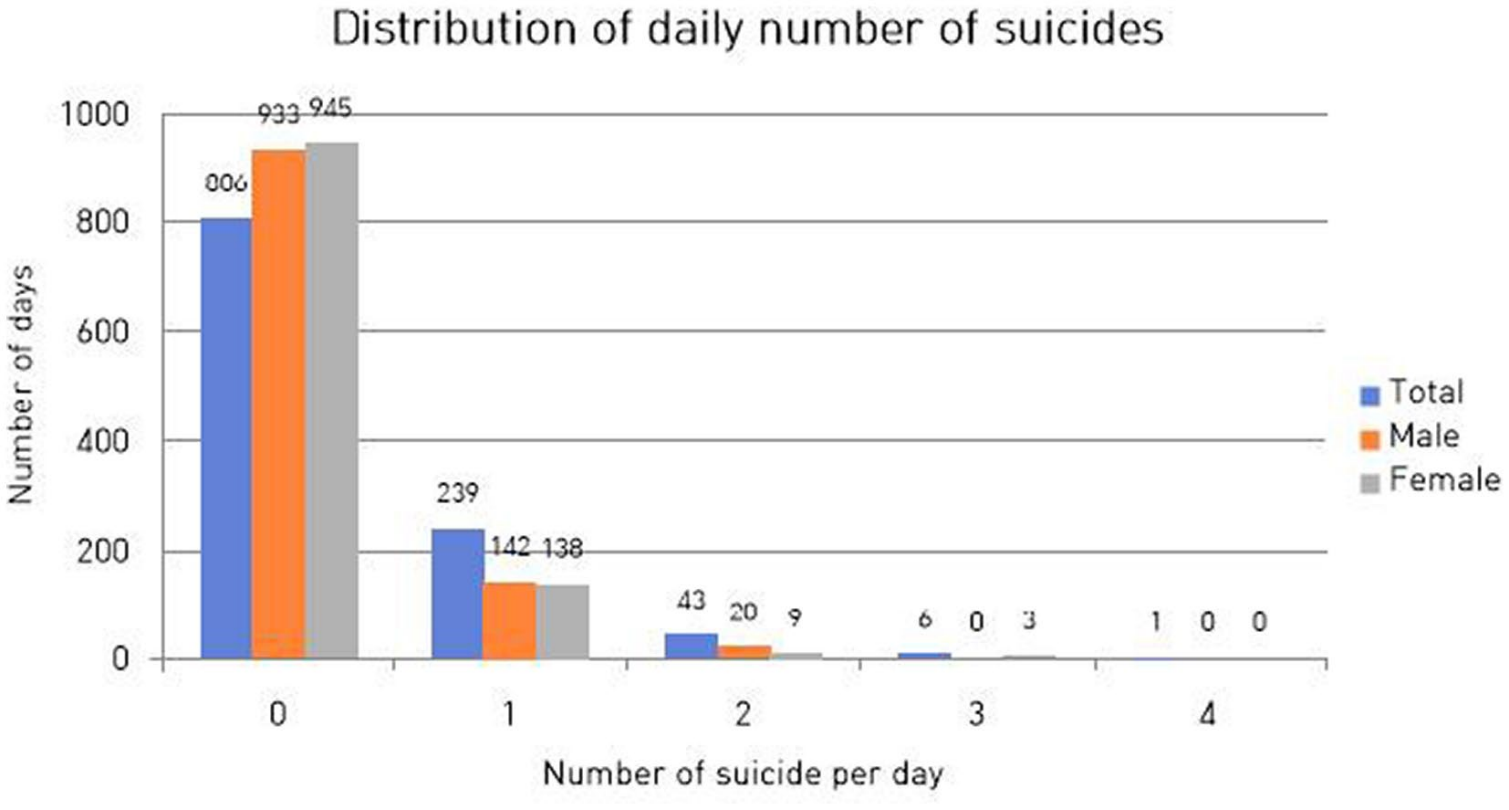
Distribution of daily number of suicides. The horizontal axis represents the number of suicide events per day and the vertical axis represents the number of days during the analysis period (1,095 days) with the corresponding number of suicides. For example, in adolescent men that are depicted with the red bar, 993 on the vertical axis indicates that for 933 days during the study period, zero suicides were documented.

The distribution of the number of suicides per day is presented in Figure 2. From January 1, 2016, to December 31, 2018, the total number of suicides was 347 (182 deaths of male adolescents and 165 deaths of female adolescents).

### 3.3. Poisson regression analysis between suicide numbers and search terms

In Table 1, we showcase the results of multivariate Poisson regression analysis between the number of suicides and search term volumes. Overall, the results for female adolescents were similar to those of the total population, and there were more statistically significant search terms for female than for male adolescents. More elaborately, the terms that showed a statistically significant association between the search volume and number of suicides differed by sex and time interval.

**Table 1:** Poisson regression analysis between search terms and number of suicides.

Time interval	Sex	Search terms	*B*	Standard Error	*z* value	*p* value
Daily	Male	School violence	0.026	0.013	2.042	0.041
	Female	Self-injury	0.068	0.011	6.264	0.000
		Family trouble	−0.108	0.053	−2.045	0.041
	Total population	Self-injury	0.027	0.013	2.020	0.043
		School violence	0.029	0.011	2.726	0.006
		Depression	0.152	0.077	1.965	0.049
		Family trouble	−0.118	0.042	−2.831	0.005
Weekly	Male	Academic score	0.494	0.221	2.238	0.025
		Depression	0.439	0.208	2.109	0.035
	Female	Suicide	0.034	0.015	2.213	0.027
		Self-injury	0.054	0.019	2.847	0.004
	Total population	Suicide	0.034	0.011	3.119	0.002
		Self-injury	0.035	0.015	2.325	0.020
Monthly	Female	Self-injury	0.058	0.022	2.647	0.008
	Total population	Self-injury	0.036	0.017	2.127	0.033
		School violence	0.035	0.017	2.123	0.034

^*^The analysis was conducted using only search terms with significant results on Poisson’s univariate analysis.

Daily search analysis of female adolescents revealed “Self-Injury” as the search term most strongly associated with suicide (*B* = 0.068, *p* < 0.000). The search volume of this term was significantly associated with the number of suicides at all three time intervals for female adolescents and the total population.

The search volume of “Suicide” showed a significant association with the number of suicides in female adolescents (*B* = 0.034, *p* = 0.027) and the total population (*B* = 0.034, *p* = 0.002) in the weekly analysis. Conversely, no significant associations were observed in the daily and monthly analyses.

The search volume of “Depression” showed a significant association with the number of suicides in the total population in the daily analysis (*B* = 0.152, *p* = 0.049) and in male adolescents in the weekly analysis (*B* = 0.439, *p* = 0.035).

The search volume of “Family Trouble” also showed a significant negative association with the number of suicides in female adolescents (*B* = −0.108, *p* = 0.041) and the total population (*B* = −0.118, *p* = 0.005) in the daily analysis.

### 3.4. Correlation analysis between search terms

Table 2 shows the correlations between the daily RSVs of each search term examined. The correlation coefficients between search term volumes were higher in female than in male adolescents. The results for the total population were similar to those for female adolescents.

**Table 2:** Correlation analysis between search terms.

Variables	Suicide	Self-injury	Depression	Academic score	School violence	Outcast	Family trouble
*Male adolescents*							
Suicide							
Self-injury	0.231^*^						
Depression	0.171^*^	0.366^*^					
Academic score	0.186^*^	−0.067^*^	−0.01				
School violence	0.011	0.003	−0.024	0.386^*^			
Outcast	0.129^*^	−0.269^*^	−0.136^*^	0.341^*^	0.202^*^		
Family trouble	0.053	−0.113^*^	0.03	0.125	0.041	0.252^*^	
*Female adolescents*							
Suicide							
Self-injury	0.43^*^						
Depression	0.463^*^	0.595^*^					
Academic score	0.129^*^	−0.083^*^	−0.126^*^				
School violence	0.026	−0.044	−0.103^*^	0.51^*^			
Outcast	−0.004	−0.284^*^	−0.31^*^	0.377^*^	0.233^*^		
Family trouble	0.104^*^	0.095^*^	0.134^*^	0.197^*^	0.278^*^	0.171^*^	
*Total population*							
Suicide							
Self-injury	0.373^*^						
Depression	0.401^*^	0.591^*^					
Academic score	0.172^*^	−0.091^*^	−0.139^*^				
School violence	0.013	−0.04	−0.1^*^	0.533^*^			
Outcast	0.053	−0.297^*^	−0.305^*^	0.432^*^	0.23^*^		
Family trouble	0.077^*^	0.035	0.101^*^	0.206^*^	0.212^*^	0.228^*^	

^*^p-value < 0.05.

The search volumes of “Depression,” “Self-Injury,” and “Suicide” showed positive correlations with each other. The correlation coefficients between these three terms were higher in female adolescents than in male adolescents, especially for the correlation between “Depression” and “Self-Injury” (female adolescents: *r* = 0.595 and male adolescents: *r* = 0.366).

Conversely, the search volumes of “School Violence,” “Academic Score,” and “Outcast” showed negative correlations with that of “Depression” in female adolescents and the total population. In female adolescents, the search volumes of “Academic Score,” “School Violence,” and “Outcast” were each negatively correlated with that of “Depression” (*r* = −0.126, −0.103, and −0.31 respectively). The correlation coefficients for the total population were similar to those for female adolescents.

## 4. Discussion

This study identified significant associations between the search volumes of suicide-related terms and the number of suicides using Naver Datalab and suicide data of Korean students. Female adolescents showed higher associations with suicide than male adolescents, and “Self-Injury” was found to be the most related term. More terms showed a significant association between their search volume and suicide for daily and weekly intervals than for monthly intervals. There were positive correlations between the search volumes of “Suicide,” “Self-Injury,” and “Depression,” and the correlation coefficients between search term volumes were higher in female than in male adolescents. This study is the first to analyze associations between daily search volumes of suicide-related terms and the number of suicide deaths in adolescents alone.

The search volume of “Self-Injury” was associated with the number of suicide deaths at all time intervals in female adolescents and the total population. A significant association was revealed between the “Self-Injury” search volume and suicide in female adolescents in daily, weekly, and monthly data. In contrast, no association was found between the “Self-Injury” search volume and suicide in male adolescents. This result indicates that self-harm is a risk factor for suicide (35, 36) and that the association between self-harm and suicide is stronger in female adolescents than in male adolescents (5, 37, 38).

“Self-Injury” refers to the act of harming one’s own body, without confirmed suicidal intent. Researchers differentiate and define self-injury with suicidal intent as nonsuicidal self-injury (NSSI) (39, 40), and NSSI is a well-kown risk factor for suicide (41). In a Swiss study, 50.6% of people who engaged in self-harm had suicidal intent (42), while in a Korean study, one-third of Korean adolescents who engaged in self-harm had suicidal intent (43). In this study, the term “Self-Injury” was used rather than NSSI beacause our primary interest was to evaluate the search terms used by Korean adolescents during the use of an Internet search engine.

To our knowledge, the relationship between the “Self-Injury” search volume and suicide death has not been previously studied. This may be because previous studies did not include “Self-Injury” in their suicide-related search terms. Although our results do not indicate a causal relationship between “Self-Injury” searches and suicide, they suggest that the suicide rate can increase rapidly with increased “Self-Injury” search volumes. It should also be noted that the time interval between the online search and the suicide was less than 1 month, which is shorter than the intervals documented in previous studies and could even bealmost simultaneous. To that extend, the development of a real-time online system that monitors the search volume of suicide-risk terms could be a useful tool to prevent adolescent suicide.

It is also important to note that the results of our study reflect the trend in 2018, a period in which adolescent self-injury emerged as a social problem in Korea, as mentioned above. Although no study has revealed the causality of this insight, several hypotheses have been suggested. Among those, in 2018, several self-injury-themed songs were popular among youths (44), and numerous self-injury-related photographs and videos were posted on social media platforms; accordingly, uploading self-injury images as a proof shot became a kind of means of communication (45). In addition, the number of adolescent patients visiting hospitals for self-injury increased rapidly. The stronger association between self-injury and suicide in female than in male adolescents (5, 37, 38) may explain the higher female suicide.

In the weekly analysis, the search volume of “Suicide” was positively associated with suicide in female adolescents and the total population. Previous studies (14, 15, 18–23) analyzed the association between the “Suicide” search volume and suicide at monthly or annual intervals. Many studies (14, 18, 20, 21, 23) have shown a significant association between the “Suicide” search volume and suicide, with some reporting a positive association (14, 18, 20, 21) while one has documented a negative association (23). The current study showed that among adolescents, online searches using the term “Suicide” were associated with suicide within 1 month, which is much shorter than the interval reported in the previous studies. Adolescents with SI may obtain a preferred suicide method through their Internet sources and find people with similar thoughts. Thus, the first result screen that runs after an online search using the term “Suicide” on Naver in Korea contains a phone number or website where the individual can find assistance, along with the phrase “You are a precious person.” Additionally, unlike other suicide-related words, “Suicide” cannot be searched on Naver blogs and cafes. In the future, it may be necessary to provide more individualized and effective suicide prevention information to individuals searching for suicide-related terms on the Internet using artificial intelligence or big data analysis.

The time interval between suicide-related term searches and suicide death is an interesting subject because it can estimate the optimal time for suicide prevention. In our study, the time interval significantly differed according to the suicide-related term. Additionally, this time interval was less than 1 month for most of the evaluated terms, which is less than those that have been reported by previous studies. We attempted a quarterly analysis, but the results of monthly and weekly analyses were less significant, accompanied by a relative small sample size. However, in case of “Suicide,” a significant association between suicide and search volume in the quarterly analysis was also found in female adolescents (*B* = 0.135, *p* = 0.001). Many previous studies (14, 15, 18–23) have shown a significant association between the “Suicide” search volume and actual suicide using monthly and yearly suicide data. Additionally, online searches with the term “Suicide” seem to have a longer time interval to suicide than the ones with the term “Self-Injury.” Accordingly, further studies with longer periods of analysis are required.

This study indicates that search terms related to suicide differ according to sex. In both the Poisson’s and correlation analyses, the results for the total population and female adolescents were similar. More search term volumes related to suicide were observed in female adolescents, and there were different correlations between search terms by sex. This finding is consistent with previous research findings (46), indicating that women tend to use the Internet more than men for health and lifestyle purposes. This suggests that suicide prevention policies based on Internet searches may be more effective for female than for male adolescents.

The correlation analysis showed positive correlations between the search volumes of “Suicide,” “Self-Injury,” and “Depression” in both male and female adolescents, with a stronger correlation in female adolescents. This finding is consistent with those of previous studies (5, 37, 38), indicating a stronger association between suicide and self-harm in women. Moreover, the search volume of “Depression” showed negative correlations with the search volumes of “School Violence,” “Academic Score,” and “Outcast” in female adolescents. This finding contrasts with those of a previous study (47) that found a relationship between “Depression” searches and searches related to academic scores, bullying, and school violence. This suggests that female adolescents searching for “Academic score,” “School Violence,” or “Outcast” in Korea may not be in a depressed state; rather, they may be actively attempting to solve these problems. Further research with longer analysis periods and additional search terms are required to clarify these differences.

In addition to “Suicide” and “Self-Injury,” other search terms related to suicide death differed by sex. In male adolescents, the search volumes of “Academic Score” and “School Violence” were positively associated with suicide in the weekly and daily analyses. Since male adolescents desire greater social recognition and have a greater desire for achievement than female adolescents (48), there may have been more associations between the “Academic Score” search volume and suicide in male adolescents. School violence in Korea has the highest proportion of physical violence, and male adolescents are exposed to physical violence to a greater extent than female adolescents (49). These factors may contribute to the association between the “School violence” search volume and suicide in male adolescents. However, in female adolescents, the “Family Trouble” search volume showed a negative association with suicide in the daily analysis. Female adolescents tend to ask for help more frequently than male adolescents (50), and female adolescents who search for “Family Trouble” may be proactively trying to solve problems rather than being depressed. However, since the search volumes for “Academic Score,” “School Violence,” and “Family Trouble” were relatively small compared to that for “Suicide,” and these terms have diverse meanings, the interpretation of these events needs to be reserved for further analysis.

A significant association was found between the “Depression” search volume and suicide in the weekly analysis only in male adolescents, despite the higher prevalence of depression in female adolescents. This is similar to the findings of a previous study (19), which demonstrated an association between the “Depression” search volume and suicide in male Internet users. Given this significant association it is important to educate and inform male adolescents on the potential risk of suicide.

Finally, a recent study (51) has shown that “Demoralization” is also a risk factor for suicide. This concept refers to a state of loss of faith in one’s expectations and abilities due to difficulties and stress in life (52); “Demoralization” resembles depression but constitutes a separate concept. A significant portion of adolescents who search for “Depression” may be in a state of “Demoralization,” and future research regarding adolescent suicide should include this concept.

## 5. Strengths and limitations

The strength of this study is that daily data were analyzed by combining Korean student suicide data with representative search engine data. Further, the data included only adolescents, and sex differences were investigated. This allowed for a more detailed time interval analysis of the association between Internet searches of suicide-related terms and the number of suicide deaths.

However, this study had several limitations. First, the sample size was limited, and data were collected over only 3 years. Second, we selected only seven suicide-related search terms and excluded subterms. In addition, this study included only adolescents who died of suicide and attended school. Additionally, when extracting information on the number of search terms, we only aggregated information relevant to the time that the adolescents were logged in. Moreover, we did not compare the association between search volume and the number of suicides according to the search method (PC versus mobile device). Lastly, this study did not adjust for factors such as celebrity suicides, suicide-related media reports, economic conditions, or suicide prevention policies that could influence adolescent suicide.

## 6. Conclusion

This study is the first to show that the search volume for “Self-Injury” in a search engine is associated with suicide death in adolescents and that the time interval from the search to suicide is less than 1 month. Further studies with a larger sample size and more search terms should be conducted to establish an online system that can detect suicide risk in adolescents and provide valuable interventions.

## Data availability statement

The original contributions presented in the study are included in the article/supplementary material, further inquiries can be directed to the corresponding author.

## Author contributions

HH, JS, and JH: conceptualization. JH and SK: formal analysis. HH: funding. HH and W-SC: supervision. JS: writing—original draft. HH, JS, and W-SC: writing—review and editing. All authors contributed to the article and approved the submitted version.

## Funding

This work was supported by the Ministry of Education of the Republic of Korea and the National Research Foundation of Korea (NRF-2018S1A5B8A02081988).

## Conflict of interest

The authors declare that the research was conducted in the absence of any commercial or financial relationships that could be construed as a potential conflict of interest.

## Publisher’s note

All claims expressed in this article are solely those of the authors and do not necessarily represent those of their affiliated organizations, or those of the publisher, the editors and the reviewers. Any product that may be evaluated in this article, or claim that may be made by its manufacturer, is not guaranteed or endorsed by the publisher.

## Footnotes

^1^ http://www.naver.com

^2^ https://datalab.naver.com/

^3^ https://datalab.naver.com/keyword/trendSearch.naver
